# Enthesitis: Much More Than Focal Insertion Point Inflammation

**DOI:** 10.1007/s11926-018-0751-3

**Published:** 2018-05-30

**Authors:** Abdulla Watad, Richard J. Cuthbert, Howard Amital, Dennis McGonagle

**Affiliations:** 1 Department of Medicine ‘B’, Zabludowicz Center for Autoimmune Diseases, Sheba Medical Center, Tel-Hashomer, Ramat Gan, Israel; 2 Sackler Faculty of Medicine, Tel-Aviv University, Tel-Aviv, Israel; 3 Section of Musculoskeletal Disease, Leeds Institute of Molecular Medicine, University of Leeds, NIHR Leeds Musculoskeletal Biomedical Research Unit, Chapel Allerton Hospital, Leeds, UK

**Keywords:** Enthesitis, Entheses, IL-17, IL-23, IL-12, JAKi

## Abstract

**Purpose of Review:**

Recognition of the importance of enthesitis as the pivotal pathological process underpinning spondyloarthropathies (SpA) has increased in recent years. Thus, we summarized the current knowledge on the pathogenic role of enthesitis on SpA shown by both animal models and human studies in vivo.

**Recent Findings:**

Experimental models have shown several SpA-like diseases that commence at entheses and are linked to nail disease as well as dactylitis, two important entheseal-associated conditions in humans. Frequently, enthesitis is not the primary outcome measure in studies of peripheral PsA and SpA although arguably it is the key parameter being indirectly assessed in spinal disease in ankylosing spondylitis. The use of different agents including JAK, IL-17, and IL-23 inhibitors contributes significantly to our understanding of enthesitis in terms of involved immune pathways.

**Summary:**

Enthesitis and enthesis organ inflammation may be the primary pathological process underlying SpA associated skeletal inflammation. Emergent studies are beginning to elucidate the molecular basis for this type of joint inflammatory response.

## Introduction

In the last decades, great advances have occurred in the etiopathogenetic understanding of the spondyloarthropathies (SpA) which was paralleled by the introduction of the TNFi therapies resulted in better clinical outcomes. A pivotal component of these advances was the recognition that enthesitis, defined as inflammation of tendon, ligament, and joint capsule insertions to the bones, is the cardinal pathological process in the SpA group of diseases [1, 2]. In this article, we focus on recent aspects of enthesis biology in experimental studies and in man and update the reader on new therapy developments for enthesitis.

## Enthesis Microanatomy

The enthesis organ, defined as a group of tissues including fibrocartilages, bursa, fat pad, adjacent trabecular bone networks, deeper fascia, and enthesis, functions collectively to carry out a common task, namely anchorage and stress resistance [3, 4]. The recognition that the enthesis is an organ helps to conceptualize why entheseal inflammation may be associated with diffuse extracapsular swelling in addition to synovitis and osteitis. An especially important component of the enthesis organ is the synovio-entheseal complex where bursal and other synovial cavity resident macrophages provide lubrication, nourishment, and metabolic requirements as well as micro-debris waste disposal in health [5]. However, these resident macrophages have also been suggested to trigger severe joint swelling in disease [3, 6–8]. The functional anatomy of the enthesis and how fibrocartilage-lined joints, some of which wrap around tendons, function like entheses and the unifying mechanical explanation for disease that stems from these concepts has been well covered previously [4, 6]. Given the fact that both the nail and the flexor tendons are respectively anchored to the skeleton via the distal interphalangeal joint tendon or accessory pulleys, creating an enthesis-like structure provides further support for the key importance of the enthesis in SpA-related skin and joint manifestations [9].

## The Primacy of Enthesitis in Animal Models

Recently, several animal model studies have demonstrated the essential role of enthesitis in pathogenesis of the SpA group of diseases [10] and shed light on the importance of specific inflammatory pathways and various cytokines in acting locally such as IL-23 [8], IL-17, and IL-22 as key pro-inflammatory cytokine in SpA potentially secreted by entheseal resident cells [8, 11]. Indeed, as result of better understanding of the immune pathways involved in enthesitis, we are witnessing novel treatment advances that offer new opportunities to improve clinical entheseal disease including IL-23 and IL-17 blockers as biological agents, and small molecules such as PDE4 inhibitors and JAK inhibition [12–14].

However, the basis for the reported PDE4 inhibition efficacy for enthesitis, but not synovitis in RA, is not understood at this time.

In the last few years, several experimental animal models of SpA-like disease have helped elucidate the role of enthesitis as the primary pathological process in SpA, identifying various entheseal T cell subtypes and new immune pathways (Table 1). Systemic overexpression of IL-23 using hydrodynamic injection of IL-23 minicircle DNA activated IL-23R+, ROR-γt+ CD3+ CD4− CD8−, stem cell antigen 1 (Sca1)+ entheseal resident T cell lymphocytes inducing the transcript expression of IL-6, IL-17, and IL-22 as well as of CXCL1 genes [8]. The same technology induced arthritis and osteoblast-mediated bone remodeling and resulted in SpA-like bone formation manifestation of SpA with IL-22 but not IL-17A DNA minicircles [8]. However, in the aforementioned model, enthesitis was IL-17A dependent and this is consistent with the SKG mouse model of enthesitis [8, 15].

**Table 1:** Selected murine enthesitis models

Reference	Species	Strain	Intervention	Characteristics
Reihardt et al. [16]	Mice	Tcrd-H2BeGFP mice crossed with mice of the susceptible B10.RIII background	Hydrodynamic injection of IL-23 minicircle DNA	Activated Vγ6+CD27− γ/δ T cells were found in uninflamed entheseal tissue and constituted the largest resident T cell subset.
Armaka et al. [59•]	Mice	TNF-overexpressing mouse model (TNF^ΔARE/+^)		Spondyloarthritis with a CD-like pathology localized primarily in the small intestine. Additionally, the development of arthritis was dependent on TNF receptor I (TNFRI) expression with mesenchymal cells being primary responders.
De wilde et al. [24]	Mice	A20^myelKO^ mice	A20 knockout	Enthesitis was found to be an early inflammatory lesion in A20^myelKO^ mice. A20 negatively modulated STAT1-dependent gene transcription in myeloid cells which was JAK/STAT dependent.
Benham et al. [15]	Mice	BALB/c ZAP-70(W163C)-mutant (SKG) mice	β-1,3-Glucan injected intraperitoneally	In curdlan-treated SKG mice, arthritis, enthesitis, and ileitis were IL-23 dependent. Enthesitis was specifically dependent on IL-17A and IL-22. IL-17A was pathogenic, while IL-22 was protective against ileitis.
Sherlock et al. [8]	Mice	B10.RIII mice	Immunization with type II collagen	IL-23 is essential in enthesitis and acts on previously unidentified IL-23 receptor (IL-23R)+, RAR-related orphan receptor gt (ROR-gt)+CD3+CD4–CD8–, stem cell antigen 1 (Sca1)+ entheseal resident T cells which led to the induction of IL-6, IL-17, and IL-22 as well as of CXCL1 secretion leading to osteoblast remodeling which is characteristic of enthesitis.

In 2016, it was shown that the majority of IL-23R-responsive cells in the normal murine entheses were γδ T cells expressing the RAR-related orphan receptor γt (ROR-γt) transcription factor, as well as the IL-23 and CCR6 receptors, and are capable of IL-17A production [16]. More specifically, Reinhardt et al. analyzed entheseal lymphocytes from C57BL/6, Tcrd-H2BeGFP, Rorc-GFP, and IL-23R-eGFP murine models, after performing hydrodynamic injection of IL-23 minicircle DNA [16]. Activated Vγ6+CD27− γ/δ T cells constituted the largest T cell subset [16]. Of particular relevance to the SpA concept was γδ T cells in the murine ciliary body, thus offering a micro anatomical and immunological link between the enthesis and the eye [17].

TNF plays a key role in the pathogenesis of IBD, in which enthesitis is also a typical manifestation when the disease also involves extra-intestinal structures [18]. Another animal model demonstrating the involvement of entheses is the TNF overexpression model that was reported by Armaka et al. [19] who used a TNF transgenic model known as the TNF^ΔARE/+^ mutant mice, characterized by the development of Crohn’s-like IBD and SpA-like disease [20]. Bone marrow-grafting experiments showed that development of arthritis was dependent on TNF receptor I (TNFRI) expression [21]. Indeed, it was shown that TNFRI expression in mesenchymal cells resulted in a SpA and intestinal phenotype, demonstrating that mesenchymal cells are primary targets in this model [19]. A confirmation of these findings is well given in the study by Milia et al. [22], who studied transgenic rats with high expression of HLA-B27 and human β (2)-microglobulin (B27TR), randomly assigned to TNFi treatment. Early and late administration of anti-TNFα antibodies prevented and improved inflammation and joint remodeling, respectively, preserving the enthesis organization [22]. Interestingly, SMAD1/5/8 signaling, a marker of bone remodeling, was not inhibited by late anti-TNFα treatment [22]. These animal models kindled considerable interest in the theory that enthesitis therapy might be associated with irreversible new bone formation lesions following biological agent therapy. At the present time, the consensus is that the earlier that biological therapy is used in man, then the lower the likelihood of entheseal new bone formation occurrence in the spine [23].

Furthermore, enthesitis, or more specifically synovio-entheseal complex disease, has been reported as an early feature of paw inflammation in mice with a myeloid cell-specific A20 (TNFα-induced protein 3) deficiency [24]. These mice are characterized by high levels of inflammatory cytokines, leading to consistent NF-κB activation and significant production of TNF, IL-1, and IL-6 by macrophages [25]. Nevertheless, in the myeloid A20 knockout mice, SpA-type arthropathy is independent of TNF but dependent on IL-1 and IL-6 [25]. In the early phase of arthritis of these mice, Achilles tendon region swelling was noted, and hematoxylin-eosin staining showed inflammation of the synovio-entheseal complex (SEC) [24]. Disease was blocked by the tofacitinib administration which provided pre-clinical support for JAKi in PsA and SpA.

The importance of the IL-23/17 axis has been further supported by other experimental studies where disease also commences at the enthesis and is associated with other SpA manifestations including nail disease and dactylitis [15, 26, 27]. There is evidence for local IL-23 production in the gut [28] and it has been proposed that systemic circulation of IL-23 could then trigger SpA at the entheses [8]. IL-23 activating resident T cells within the enthesis promotes inflammation and bone remodeling mediated by IL-17 and osteoproliferation mediated by IL-22 [29]. Thus far, there is little data on the source of IL-23/17 axis cytokines driving human arthritis and questions relating to whether IL-23 in particular is locally or systemically produced remain to be elucidated.

## Human Studies of the Enthesis

**Fig. 1:**
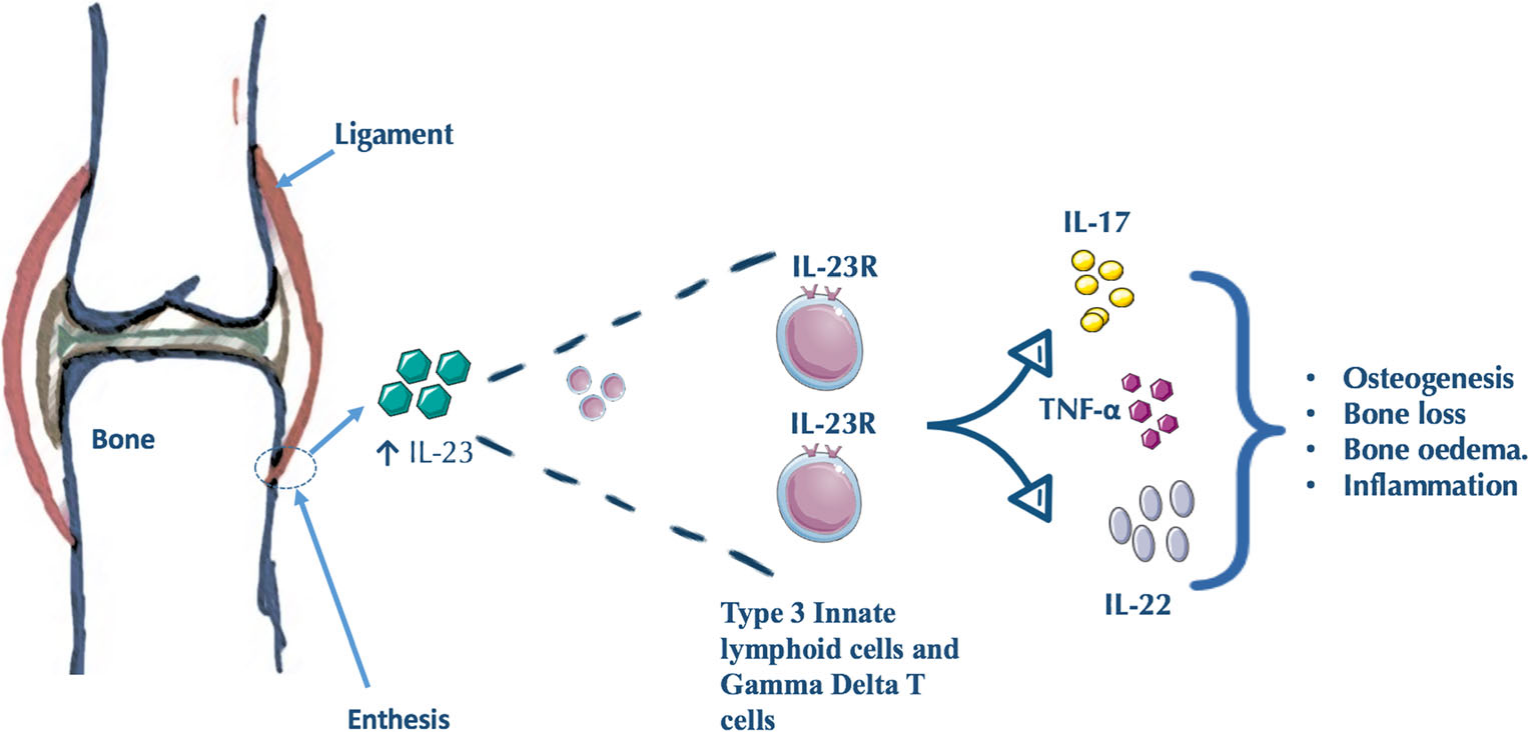
Lymphocyte populations defined at the human enthesis. Thus far, two lymphocyte populations have been defined at the human enthesis. Innate lymphoid cells are part of the IL-23-responsive T cells which are residents of the healthy enthesis. Gamma delta T cells are also resident at the enthesis. The activation of resident T cells within the enthesis by IL-23 may promote inflammation, osteogenesis, and bone loss and remodeling. These lymphocyte populations may release different cytokines including IL-17 and IL-22 and TNF-α

The description of an IL-23-responsive population of T cells [8], along with the description of a group of cytokine-dependent innate lymphoid cells (ILCs) [30], led to a search for innate immune cells at the normal human enthesis. We selected small normal interspinous process entheses from donors with no systemic inflammatory disease to explore the presence of type 3 ILC populations. [31••]. The small interspinous process entheses have advantages over large attachment sites like the Achilles tendon where thick juxta-articular fibrocartilaginous tissue that is completely devoid of immune cells precludes immune cell subset evaluation. We showed that the normal human enthesis did indeed harbor a rare population of IL-23R-expressing type 3 ILCs [31••]. We were also able to demonstrate the presence of a comparatively abundant population of γδ T cells. In health, entheseal cells were present in both the enthesis soft tissue and the peri-entheseal bone [31••]. Furthermore, there was little evidence for expression of IL-17A transcripts in health, but of note, the IL-23R+ type 3 ILCs expressed TNFα transcript [31••]. Following stimulation of human entheseal tissue with IL-23 and IL-1β, we demonstrated upregulation of IL-17A and evidence for upregulation of IL-17F and IL-22 (Fig. 1). The inducible expression of IL-22 on these entheseal resident immunocytes and the recognition that IL-22 drives human MSC osteogenesis [32•] fit into a model whereby inflammation at the entheses may later drive new bone formation (Fig. 1).

## Evaluating Enthesitis

Unfortunately, in humans, enthesitis remains stubbornly difficult to assess [33]. The complete avascularity of entheses at bone attachment sites and the low vessel density in adjacent ligaments and tendons as well as the lack of adaptive hyperplasia that is normally seen in synovitis make assessment of these sites particularly challenging [33]. Moreover, despite the existence of various clinical measures for enthesitis, there is no sound consensus concerning their validity in PsA, since gold standard validation has not been performed. Therefore, it is necessary to rely on imaging rather than clinical examination in order to detect non-accessible sites of entheses. Indeed, numerous studies have shown that ultrasound (US) could detect a subclinical enthesitis in PsA patients [34–36].

**Fig. 2:**
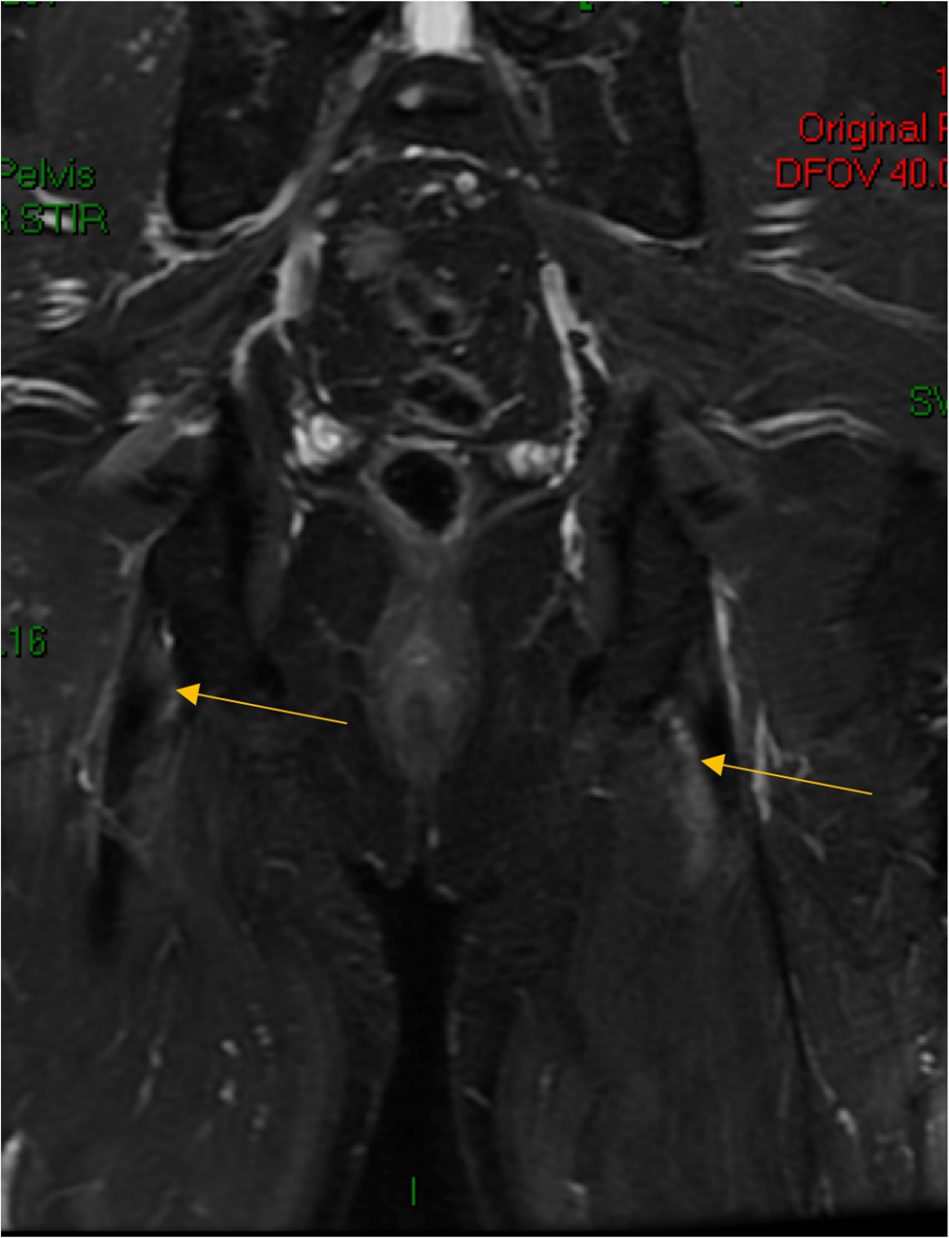
Magnetic resonance imaging (MRI) of the sacroiliac joints (SIJ) showing bilateral ischial tuberosity enthesitis in a patient with newonset PsA. The site of soft tissue entheseal inflammation is depicted by arrowheads. In this case, there is sparing of the bony attachment.

**Fig. 3:**
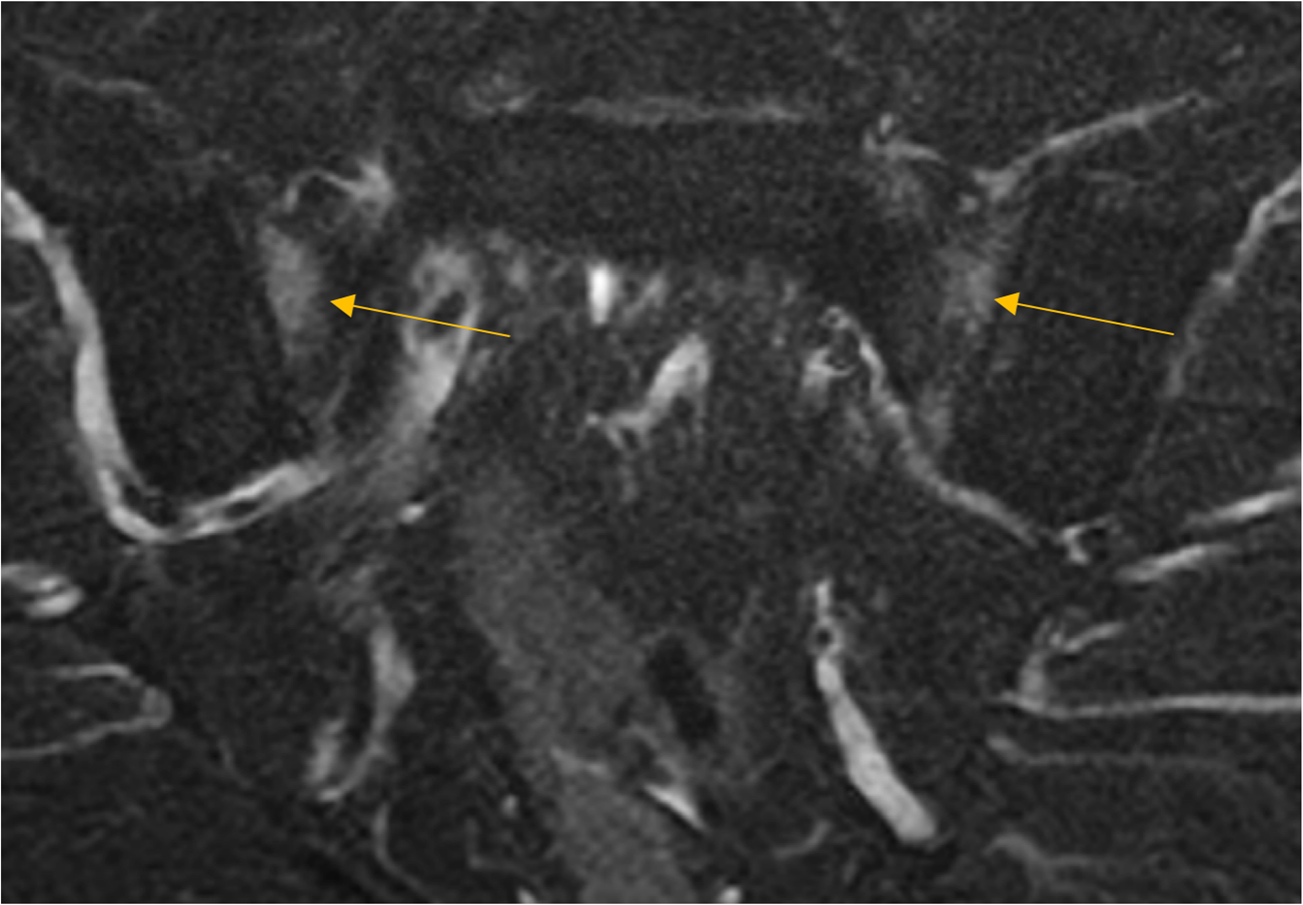
Magnetic resonance imaging (MRI) of the sacroiliac joints (SIJ) showing bilateral sacral joint bone marrow edema on in a patient with early psoriatic arthritis. The Bone marrow edema is more florid at the anterior part of the joint adjacent to the capsular enthesis. It remains to be determined whether the patterns of inflammation at entheses in soft tissue or in the bone may influence responses to therapy

Typically, enthesitis is associated with diffuse peri-entheseal soft tissue edema on magnetic resonance imaging (MRI) with this adjacent reaction being usually more conspicuous than inflammation within the insertion which reflects the greater vascularity of the peri-entheseal tissues (Fig. 2). Both entheses and sacroiliac fibrocartilaginous joints have prominent fibrocartilages and disease of both structures may be associated with an adjacent severe osteitis that likely reflects the excellent vascularity of the marrow (Fig. 3). However, neither MRI nor US excludes the presence of enthesitis, and the role of imaging remains controversial since the use of power Doppler (PD) which is typical of RA-related synovitis activity is generally much less conspicuous at insertions [37, 38]. Studies comparing clinical enthesitis with imaging enthesitis in SpA show virtually no correlations [34]. Furthermore, where suspected pathology is seen, it is uncommon to get tissue to confirm the diagnosis. Therefore, the assessment criteria remain subjective. The exact histopathological basis for the subclinical entheseal abnormalities present in PsA and other forms of SpA is poorly understood. However, a recent US study reported in abstract form has shown that subclinical enthesopathy in psoriasis cases regresses following anti-IL-12/23 therapy, suggesting an inflammatory component [39].

Among the diverse clinical measures for enthesitis, the Leeds Enthesitis Index (LEI) uses the medial collateral ligament (MCL) origin in both knees as a site of enthesitis [40]. This anatomic structure forms an elaborate synovio-entheseal complex that might be inflamed as a result of events taking place elsewhere within the synovial cavity; thus, it is hard to interpret exactly what tenderness at this location means and whether it could be linked to synovitis. It might be that these difficulties and complexities in recognizing and measuring enthesitis contributed to the GRAPPA-OMERACT for PsA standardization unwittingly dropped enthesitis from core domain assessments [41]. The move towards trials in early PsA makes the need to address enthesitis an urgent one since early disease may might be strongly linked.

## Recent Clinical Developments

The response of human enthesitis to various therapeutic agents goes some way to improve the rudimentary state of immunological knowledge about human enthesis and the diverse immune pathways that are involved in disease [42]. Moreover, the use of enthesitis as an important manifestation of PsA, and its evaluation in drug trials, and clinical practice as well, has increased dramatically with enthesitis now a universal secondary outcome measure in trials [43]. The treatment of enthesitis remains a challenge. Conventional DMARDs such as sulfasalazine and methotrexate are not significantly effective for enthesitis management, yet NSAIDs remain the first-line therapy [43] although corticosteroid therapy including injection, where appropriate or feasible, is also an option. TNF blockade is the standard of care for enthesitis and severe axial disease in those patients with partial response to conventional DMARDs [44]. However, in recent years, many efforts were made to assess the efficacy of several therapeutic agents in enthesitis.

Recent phase 3 studies have shown efficacy of PDE4 blockers for PsA but with lower ACR 20 responses compared to the biologic class of drugs. A favorable effect of the PDE4 blocker, apremilast, on enthesitis has been reported [45, 46]. Given that the PDE4 blockers are ineffective in RA and do not block autoantibody production but suppress the neutrophil influx to sites of tissue inflammation [47], it is likely that these agents are working predominantly on innate immunity. This supports the idea of what we termed MHC-1-opathy [48], whereby in the SpA group of diseases, initial site-specific innate immune activation drives adaptive immune activation via CD8+ T cells, which in turn drive IL-17-related neutrophilic tissue inflammation exacerbation of these diseases [48]. Two recent studies have evaluated Janus kinase (JAK) inhibition with tofacitinib, a pan JAK inhibitor for the treatment of PsA [49•, 50]. In biologic naïve cases, tofacitinib was compared to adalimumab, where enthesitis was assessed as a secondary outcome, reporting a favorable response [50]. However, in the second study, the pre-specified statistical hierarchical model used precluded an evaluation of enthesitis [49•]. It is likely that JAK inhibition will represent a new option in the treatment of PsA and enthesitis. The precise mechanism of action of JAK inhibition is unclear since over 50 different cytokines including interleukins, CSFs, and hormones that share various signaling pathways are mediated by JAKs [51••].

However, the two biggest classes of drugs used to treat PsA and AS, namely TNF and IL-17 inhibitors, do not signal directly through the JAK pathway [52]. Nevertheless, IL-23 signals via tyrosine kinase 2 (Tyk2) blockers for psoriasis support the idea of a therapy effect via IL-23 alone [51••, 53].

An unexpected finding in this field is the apparent non-efficacy for ustekinumab for spinal enthesitis of AS despite its efficacy in the peripheral skeleton (data not published). The most obvious avenues for exploration are that the immune system at the immunobiology peripheral and axial entheses may be fundamentally different and that higher doses of anti-IL-23 pathway cytokine are needed for axial disease. One obvious difference is the relative absence of the synovio-entheseal complex in the spine and where pathology is often located at entheseal bone anchorage points [33]. However, this intriguing observation around IL-12/23 blockade apparent non-efficacy awaits further study including data on the IL-23 pathway antagonism with specific p19 blockers.

The IL-17 blockers have also been evaluated for their effect on enthesitis. Secukinumab a IL-17A blocker has shown efficacy for enthesitis in several trials [54, 55]. Ixekizumab, a second IL-17A blocker with a 50-fold higher affinity, showed efficacy for enthesitis in the P-SPIRIT-1 study [56]. However, in the P-SPIRIT 2 study with the same molecule in PsA cases that failed to respond to prior TNF blockade, there was no significant effect on enthesitis at the pre-specified 24-week assessment, but there was evidence for efficacy at earlier time points [57]. A third molecule, brodalumab, an IL-17RA blocker, was also evaluated for its effect on enthesitis and no significant effect was found in those patients who had scores of more than 0 for enthesitis at week 12 between the group receiving 140 mg of brodalumab and the group receiving 280 mg of brodalumab, as compared with the placebo group [58]. However, although not statistically significant, there was a greater numerical improvement in enthesitis in subjects on higher dose of brodalumab. Given all the genetic and pathological data and the existing studies, it seems that these agents will be effective for the therapy of enthesitis.

## Conclusions

Enthesitis is much more than local inflammation; indeed, it is considered as the primary pathological process underling SpA. This has been shown by investigating the enthesis in both animal models and human studies in vivo. Enthesitis management is still a challenging matter for rheumatologists and various new agents were evaluated for their effect on enthesitis reporting different findings. The use of different agents including JAK, IL-17, and IL-23 inhibitors have contributed significantly to our understanding of enthesitis in terms of involved immune pathways. Nevertheless, there is an unmet need for further studies to improve our understanding about enthesopathy seeking a better detection, and therefore management.
